# Accurate Prediction of Protein Tertiary and Quaternary Stability Using Fine-Tuned Protein Language Models and Free Energy Perturbation

**DOI:** 10.3390/ijms26157125

**Published:** 2025-07-24

**Authors:** Xinning Li, Ryann Perez, John J. Ferrie, E. James Petersson, Sam Giannakoulias

**Affiliations:** 1 Department of Chemistry, University of Pennsylvania, Philadelphia, PA 19104, USA; xngli@sas.upenn.edu (X.L.); ryper@sas.upenn.edu (R.P.); 2 Division for Advanced Computation, Sentauri Inc., Glenwood, MD 21738, USA; jackferrie@sentauriai.com

**Keywords:** protein language models, protein stability, protein complex stability

## Abstract

Methods such as AlphaFold have revolutionized protein structure prediction, making quantitative prediction of the thermodynamic stability of individual proteins and their complexes one of the next frontiers in computational protein modeling. Here, we develop methods for using protein language models (PLMs) with protein mutational datasets related to protein tertiary and quaternary stability. First, we demonstrate that fine-tuning of a ProtT5 PLM enables accurate prediction of the largest protein mutant stability dataset available. Next, we show that mutational impacts on protein function can be captured by fine-tuning PLMs, using green fluorescent protein (GFP) brightness as a readout of folding and stability. In our final case study, we observe that PLMs can also be extended to protein complexes by identifying mutations that are stabilizing or destabilizing. Finally, we confirmed that state-of-the-art simulation methods (free energy perturbation) can refine the accuracy of predictions made by PLMs. This study highlights the versatility of PLMs and demonstrates their application towards the prediction of protein and complex stability.

## 1. Introduction

The stability of a protein or protein complex is a key parameter in development processes from bioengineering to biologics. Often, improvement of a protein’s stability is principally necessary to enhance the performance of enzymes, reduce the aggregation propensity of antibodies, and increase a protein’s half-life. Moreover, protein complex stability, the binding affinity of two proteins, is crucial for the development of specific and potent antibodies. As the number of applications of proteins continues to grow, the demand for computational tools that facilitate and accelerate the development process so too increases. To this end, we develop computational tools herein, primarily through protein language models (PLMs), that accurately predict mutational impacts on protein stability and function.

PLMs are deep learning models based on the transformer architecture. These models have been trained with a masked language objective on large corpora of protein sequences for the eventual use in fine-tuning towards particular tasks of interest. PLMs are an ideal choice for developing computational tools with maximum utility because, once they are trained, the computational time required to use such models is minimal, making them accessible to non-expert end users. Furthermore, the power of PLMs is underscored by the single requirement of a protein sequence as an input, which is far easier to obtain than structural information. In recent years, large language model (LLM) [1,2,3] methods have emerged as a transformative approach in natural language processing (NLP) [4,5], revolutionizing various tasks such as machine translation [6,7], text generation [8], and sentiment analysis [9]. By harnessing the power of transformers (the underlying architecture of LLMs), researchers have achieved remarkable capabilities in capturing long-range dependencies in sequential data, leading to state-of-the-art performance on many NLP tasks. To address the intricacies associated with protein sequences, researchers have adapted LLM techniques to create PLMs such as ProteinBERT [10], ProtBERT [11], ProtT5 [11], and ESM [12]. These models are specifically designed to capture the intrinsic relationships between amino acids by analyzing sequences alone, enabling tasks such as secondary structure prediction and subcellular location prediction, which act at the token and sequence level, respectively [13]. Numerous studies have explored the potential of fine-tuning PLMs for various downstream tasks, such as engineering protein function [14,15,16], identifying sites of post-translational modification [17], and assessing toxicity [18]. Despite the multitude of successes of PLMs, to date, the majority of methods focused on tertiary or quaternary stability prediction use structural information and simulation as inputs [19,20]. Since protein and protein complex structures are often difficult to obtain, requiring extensive amounts of time and resources, we sought to investigate the utility of PLMs for such tasks, as structures are not required.

Herein, we demonstrate that fine-tuning of a ProtT5 model on the largest protein mutant stability dataset published to date produces a highly accurate PLM capable of predicting the absolute stability of a protein from sequence alone. Furthermore, we highlight that PLMs can also capture relationships between sequence, stability, and function, by learning the impact of mutations on green fluorescent protein (GFP) brightness. Finally, we show that the utility of PLMs can be extended beyond single proteins and can successfully learn the impact of mutations on protein complex stabilities. We demonstrate that the developed PLM effectively identifies stabilizing mutations that then can be accurately quantified through more traditional, structure-based, and computationally intense, free energy perturbation (FEP) calculations. Overall, this work highlights the ability of PLMs to provide rapid and accurate predictions from mutational data on both individual proteins and complexes, predictions that can be further refined by more traditional, structural approaches.

## 2. Results and Discussion

### 2.1. Predicting Mutational Impact on Protein Stability

We selected Tsuboyama et al.’s [21] comprehensive free energy of unfolding (ΔG) dataset to initially develop a method for predicting mutational impacts on protein stability. This dataset offers an ideal setting for PLMs to showcase their abilities in such tasks, due to its high-quality, single-source data and its size, which surpasses that of other ΔG datasets by several orders of magnitude. We meticulously compiled and pre-processed the nearly one million datapoints in this dataset for machine learning (ML). Table 1 provides a summary of the data, including key metrics such as the number of unique domains and the distribution of ΔG across the various bins in all ML sets. To evaluate other characteristics of the Tsuboyama dataset, we performed exploratory data analysis and investigated the relationship between mutation type and the resultant stabilization or destabilization (Figures S1 and S2, Table S1). We determined that there were no obvious general trends beyond proline being the most consistently destabilizing type of mutation across the entire dataset (Figure S1). However, when we investigated the effects of mutations of specific amino acids within protein domains, we found diverse and specific trends. For example, Figure S2 shows an example bar chart where for a specific domain, the amino acid stabilization profile is quite different than that of the average profile across the entire dataset. This yeast Myo5 SH3 domain (PDB ID:1YP5) exhibited strong destabilization by acidic amino acids and an uncharacteristic stabilization by cysteine compared to all other types of mutations. Looking deeper, we tested to see whether certain mutations had greater or lesser effects within different secondary structural motifs. Using “define secondary structure of proteins” (DSSP) [22] to generate the secondary structure labels for each wild-type domain (or AlphaFold2 as provided in Tsuboyama et al. [21]), we computed the average effect of mutations in secondary structures both generally and in each specific domain. Generally, there does not appear to be a particular secondary structural motif sensitive to mutation, beyond B (residue in isolated β-bridge) or I (5-turn helix) type secondary structures exhibiting small destabilizing preferences (Figure S3). However, within domains (based on present motifs), there are unique and specific trends. For example, SI Figure S4 displays an example of a multi-secondary structured domain (1EKL) which exhibits preferential stabilization and destabilization. This type III antifreeze protein domain is more strongly stabilized by mutations in G (3-turn helix) and T (hydrogen bonded turn) type secondary structure while simultaneously being significantly destabilized by mutations in B, E (extended strand in parallel and/or anti-parallel β-sheet), H (4-turn helix), and S (bend) secondary structure. Together, these analyses support the idea that simple heuristics accounting for amino acid identity and secondary structure are insufficient for predicting effects on protein stability, although different or more complicated measures may be more effective.

**Figure 1:**
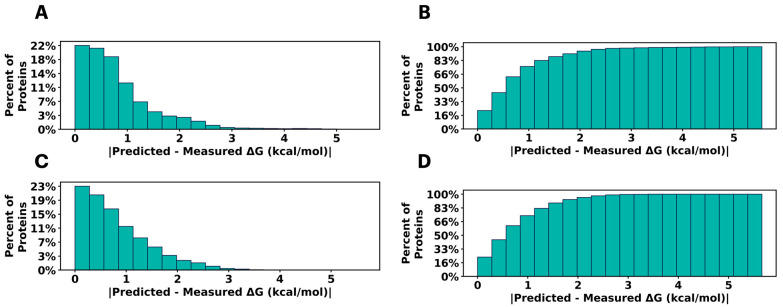
Histogram of predicted ΔG error (**A**) for the validation set, histogram of percent recovery as a function of predicted error (**B**) for the validation set. Histogram of predicted ΔG error (**C**) for the testing set, histogram of percent recovery as a function of predicted error (**D**) for the testing set.

Therefore, we investigated whether fine-tuning a PLM could enable it to learn the stabilities of different tertiary structures and how mutations alter a fold’s stability [23,24]. To do this, we fine-tuned a ProtT5 model on the Tsuboyama dataset and observed relatively strong performance in the validation and testing sets (Figure 1). Although one might suspect that the PLM, which considers the entire protein sequence, would not be sufficiently sensitive to single amino acid changes, the high correlation (R^2^ = 0.60) and low error (MSE = 1.09) for the testing set demonstrates that PLMs can effectively learn from point mutation data by simply looking at the full sequence (see Table S4 for the complete validation and test metrics). Moreover, the fact that the Tsuboyama dataset includes a plurality of domains, the PLM’s performance indicates that it also understands differences between large changes in sequence, all in the absence of structural information. Overall, these data demonstrate that this model is capable of directly predicting ΔG of unfolding from sequence alone, highlighting the utility of PLMs in predicting protein stability.

### 2.2. Predicting Mutational Impact on Protein Function

**Figure 2:**
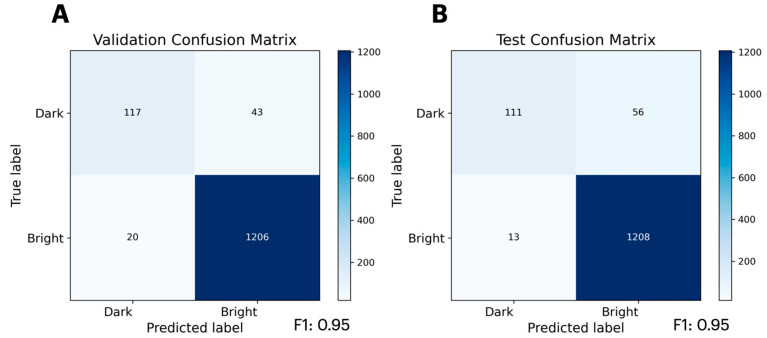
Benchmarking of the PLM fine-tuned on GFP brightness data from Sarkisyan et al. [25] Confusion matrix of GFP prediction for the validation set (**A**) and testing set (**B**).

Following our success in predicting protein stability using mutational data, we suspected that PLMs may be capable of learning mutational data from functional assays, especially if the function is directly related to protein stability. Therefore, we attempted to train a classifier from the Sarkisyan [25] GFP mutant brightness dataset, as GFP fluorescence is directly related to β-barrel stability. Furthermore, this dataset is another large, high-quality, single-source dataset, and exploratory data analysis reveals that basic heuristics based on amino acid identity and secondary structure alone cannot reliably predict the brightness of GFP mutants (Figures S5 and S6, Table S2). Rewardingly, we again observe that PLMs can accurately learn from mutational datasets using full-length protein sequences as the input, this time capturing functionally relevant information (Figure 2). The resultant classification not only has a high accuracy, but boasts a 0.95 weighted F1-score, underscoring the model’s precision and recall. Complete validation and test metrics—including weighted F1-score, weighted accuracy, weighted precision, and weighted recall are summarized in Table S5. Overall, the PLM’s ability to learn from these functional data suggests that the evolutionary information used to initially train PLMs does not overly bias the model to prevent the successful fine-tuning. The accuracy of the model also assuages concerns that a point mutation within a whole protein sequence is too minimal a change to allow PLMs to learn the impact of such mutations on a specific protein’s function.

### 2.3. Predicting Mutational Impact on Protein Complexes

**Figure 3:**
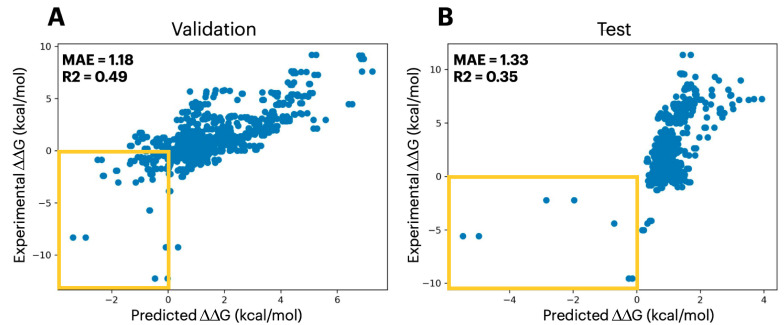
Plot showing the correlation between the predicted mutational ΔΔG values with the experimental values for the validation set (**A**) and the testing set (**B**), respectively. The yellow boxes highlight the region of stabilizing mutations.

Given that the two prior fine-tuning tasks show that PLMs can capture mutational impacts on tertiary structure stability and closely related protein functions, we sought to test whether PLMs could be extended beyond single protein systems to capture mutational impact on quaternary structure stability. To do so, we performed fine-tuning on data from SKEMPI 2.0 [26], a database containing the change in free energy of binding (ΔΔG) associated with mutations present at protein–protein interfaces. The SKEMPI 2.0 dataset provides ΔΔG values, representing the changes in binding free energy of mutant complexes relative to their corresponding wild-type affinities. Therefore, the PLM modeling process becomes more complicated as information from two sequences must be utilized. Our model handles this by accepting two sequences to the forward pass. The first of which is the WT protein sequence 1 concatenated with the WT protein sequence 2 (this represents the wildtype complex). Subsequently, the second input is the mutant protein sequence 1 concatenated with the mutant protein sequence 2 (this represents the mutant complex). Understanding that the two proteins are separate entities, we concatenated them utilizing a new special “break token” to delineate them. As anticipated, the added complexity of this dataset presented a greater challenge than the single protein mutation datasets, resulting in poorer R2 metrics of 0.35 on test set (the complete validation and test metrics are shown in Table S6). However, it is worth noting that, by the nature of the dataset, there are significantly more mutations that result in a positive ΔΔG (destabilizing the complexes) than there are mutations that produce a negative ΔΔG (stabilizing the complexes). Indeed, further inspection of the predicted and observed ΔΔG values reveals that mutations with a positive ΔΔG follow a tighter and more linearly correlated relationship than those with negative ΔΔG values (Figure 3).

Although the accuracy of the predicted magnitude of the ΔΔG associated with a mutation is limited for this PLM, the model can effectively classify stabilizing mutations from those that are destabilizing (Figure 3). To further illustrate this, we fine-tuned ProtT5 to classify stabilizing and destabilizing mutations, achieving a weighted F1-score of 0.73 on the test set; the complete validation and test metrics—weighted F1-score, weighted accuracy, weighted precision, and weighted recall—are reported in Table S7. Therefore, we envision that this PLM can be used to rapidly identify mutations that would, for example, increase the stability of a protein complex, and be paired with a downstream, more computationally intensive analysis with greater accuracy to select the mutations that provide the highest magnitude of stability. To this end, we investigated the use of the FEP method [27], which uses enhanced sampling molecular dynamics (MD) to identify the change in free energy associated with a particular alchemical transformation. As a proof-of-principle, we selected three of the seven mutations that were predicted to be stabilizing by the PLM and simulated the ΔΔG using FEP. Indeed, we observe that FEP can provide a highly accurate calculation of ΔΔG for the mutation, allowing it to act as a downstream tool for ranking mutations identified using the PLM. We expected that accurate prediction for 1CSO_I_I_18_L would be more challenging than the others because it was a small mutation from ILE to LEU. Beyond the SKEMPI 2.0 testing set, we demonstrate an example of the accuracy of FEP simulations in a common biologics design scenario, an antibody–antigen complex (Table 2 and Table S8). This approach illustrates the PLM’s utility in being able to rapidly and accurately identify stabilizing, and destabilizing, mutations, whose magnitude can be accurately predicted using more computationally expensive methods.

## 3. Conclusions

Together, these data demonstrate that accurate computational tools can be developed that predict protein tertiary and quaternary stability, critical parameters for the development of proteins for a variety of applications. Through this effort we have demonstrated ways in which PLMs can effectively predict mutational effects on protein and protein complex stabilities, despite lacking structural information previously viewed as essential for such prediction tasks. This underscores the value of PLMs, as the requirement of simply having a protein sequence, without having to consider more difficult to obtain structural data, provides a broad application potential. Furthermore, their computational efficiency, following fine-tuning, makes them highly valuable for rapidly screening the large number of sequences often present in the mutational libraries available for most protein engineering and biologics optimization. Our ability to predict the absolute stability of proteins, regardless of fold, from sequence alone underscores this method’s ability to balance both global aspects of protein sequence with residue-level information in the form of point mutations, a necessary feature for such technologies. Although our quaternary stability PLM lacked the numerical accuracy that one might hope to achieve, its ability to effectively pick out sequences that increase or decrease complex stability is highly valuable. This is especially true when one considers the performance of the more traditional FEP method, which can be used to subsequently rank stabilizing mutants [27]. Future work will focus on improving PLM accuracy for predicting stabilizing mutations through data augmentation strategies as well as exploration of newer alternative language model architectures. Overall, this work highlights the power of PLMs and showcases a set of computational tools that can be used to predict the stability of both isolated proteins and protein complexes.

## 4. Methods

### 4.1. Data Preprocessing: Protein Stability

The dataset used for training ProtT5 model from the transformers library version 4.28.1 to predict protein stability is derived from Tsuboyama et al. [21] This dataset is a mega-scale evaluation of protein mutant ΔG of folding and represents the largest database of protein mutant stability to date. The Tsuboyama dataset includes 851,552 high-quality data points made from over 1.8 million measurements of protein folding stability with orthogonal assays to assess quality. These measurements come from single and double mutations in 542 different protein domains, each of which is between 40 and 72 residues in length. However, the Tsuboyama dataset also included several mutations which were insertions or deletions. These types of mutations represent a significantly different meaning from the majority of the data and were therefore removed. Additionally, given the nature of their assay with respect to construct transcription and translation from oligonucleotide pools, some of the single or double mutations were not incorporated. For example, in an experiment attempting to measure the double mutant D26E_D28R, where the resultant construct made was actually D26E_D28D, only the first of the two mutations were successfully incorporated. Datapoints like these were also removed, leaving 699,793 datapoints for ML.

The culled Tsuboyama dataset was then split into ~80-10-10 training, validation, and testing sets where members of each set were kept in groups according to protein domain. Splitting by groups ensures that model training and evaluation are being assessed on out-of-group generalizability. The explicit enumeration of our datasets can be found on our GitHub at https://github.com/ejp-lab/EJPLab_Computational_Projects/tree/master/ProteinStability/dG, accessed on 17 July 2025.

### 4.2. Data Preprocessing: GFP Brightness

The data for training ProtT5 model from the transformers library version 4.28.1 to predict GFP brightness was derived from Sarkisyan et al. [25] In a procedure similar to preprocessing of the Tsuboyama dataset, the Sarkisyan dataset was culled to only keep single and double mutants of GFP. The culled Sarkisyan dataset was then split into ~80-10-10 training, validation, and testing sets through multi-label stratification. In each set, the ratios of two labels are kept around ~90-10 to guarantee the representativeness of datasets. When datasets were split, they were performed with a residue position-based grouping method. Splitting by position allows there to be no redundant mutation positions between sets and again forces model training and evaluation to assess out-of-group generalizability. The explicit enumeration of our datasets can be found on our GitHub at https://github.com/ejp-lab/EJPLab_Computational_Projects/tree/master/ProteinStability/GFP, accessed on 17 July 2025.

### 4.3. Data Preprocessing: Protein Quaternary Structure Stability

To train the ProtT5 model from the transformers library version 4.28.1 to predict mutational impacts on quaternary structure stability, we utilized data from the SKEMPI 2.0 database, which provides information on the change in binding free energy (ΔΔG) associated with mutations at protein–protein interfaces. To obtain the full protein sequences from the SKEMPI dataset, we aligned sequences derived from the SEQRES header info from the originally deposited PDB structures. Those sequences were aligned to SwissProt [28], to recreate the correct mutations provided by SKEMPI 2.0. For input preparation, each pair of interacting protein sequences was concatenated with a special break token placed in between, signaling that the input consists of two separate proteins.

Data augmentation was applied to create a model agnostic to the ordered input of the two protein sequences by adding training examples that were simply the reversed order of the initial paired sequences. After performing this data augmentation, we produced a final SKEMPI dataset of size 6338 points.

In a procedure similar to preprocessing of the Tsuboyama dataset, the SKEMPI dataset was split into ~70-15-15 training, validation, and testing sets, where mutational sequences of the same protein domain were kept in the same set to avoid overfitting. The explicit enumeration of our datasets can be found on our GitHub at https://github.com/ejp-lab/EJPLab_Computational_Projects/tree/master/ProteinStability/ddG, accessed on 17 July 2025.

### 4.4. Model Training

We fine-tuned both regression and classification versions of ProtT5 starting from the Rostlab checkpoint prot_t5_xl_uniref50 available on Hugging Face (transformers library version 4.28.1) [11]. The final models for each case study were selected from the best trial of Bayesian hyperparameter searching with the TPE sampler in the Optuna Python library 3.3.0 [28]. Ten trials were searched while attempting to minimize the mean squared error loss of the validation set for regression problems, and cross entropy loss for classification tasks. Models were trained for a maximum of 10 epochs with an early stopping patience of 2 epochs. Finally, the tuned models were used to predict the data of the held-out testing set. The best hyperparameters for all models are shown in Table S3.

### 4.5. Free Energy Perturbation

FEP calculations were performed in GROMACS 2024.1 using the Amber 99sb-star-ildn force field and spce water model largely adhering to the standard protocol described by others [29]. In brief, mutations were set up by aligning amino acids to one another based on their maximum common substructure, using RDKit 2023.9.1, followed by exhaustive chi angle sampling to generate non-clashed initial poses. Subsequently, dual topologies were generated from these aligned structures and equilibration and 5 ns production MD simulations were performed using the REST2 [30] enhanced sampling method across 12 lambda states. The Bennet Acceptance Ratio (BAR) (gmx_mpi bar) analysis was performed in GROMACS on the resultant trajectory xvg files to calculate the change in free energy associated with each mutation. Simulations were tested on various production run time scales for convergence in the BAR ddG, leading to the selection of 5ns.

## Figures and Tables

**Table 1:** Statistics of machine learning sets derived from Tsuboyama et al. [21] protein stability dataset.

Set	Datapoints	Domains	Bin 1	Bin 2	Bin 3	Bin 4	Bin 5	Bin 6	Bin 7	Bin 8
Training (~80%)	546,550	432	5.41	6.58	18.53	22.54	22.02	16.27	7.51	1.14
Validation (~10%)	74,169	55	6.92	6.47	16.82	24.50	22.81	14.41	7.33	0.74
Testing (~10%)	79,074	55	8.33	9.28	17.02	20.64	21.36	15.13	7.46	0.78

Each % Bin column lists the percentage of the training, validation, or testing set that falls within a particular ΔG of unfolding range. % Bin1, % Bin2, % Bin3, % Bin4, % Bin5, % Bin6, % Bin7, and % Bin8 list the percentage of data that falls within ΔG of unfolding ranges of ΔG < −1, −1 < ΔG < 0, 0 < ΔG < 1, 1 < ΔG < 2, 2 < ΔG < 3, 3 < ΔG < 4, 4 < ΔG < 5, and ΔG > 5 in kcal/mol.

**Table 2:** FEP prediction of protein complex ΔΔG data from SKEMPI *.

Mutant	Experimental (kcal/mol)	FEP (kcal/mol)	PLM (kcal/mol)	FEP Error (kcal/mol)	PLM Error (kcal/mol)
1B2U_A_A27K	−5.03	−6.68	0.194	1.65	5.22
1B2U_A_A27K_D_A36D	−9.56	−10.90	−0.195	1.34	9.37
1CSO_I_I_18_L	−4.41	−0.40	−0.189	4.01	4.22

* Mutants are listed as their PDB accession, the chain(s) where a mutation(s) takes place, and the mutation, each separated by an underscore.

## Data Availability

The data used to train ProtT5 to predict protein stability, GFP brightness, and protein quaternary structure stability including all training, validation, and testing splits are available on our GitHub at https://github.com/ejp-lab/EJPLab_Computational_Projects/tree/master/ProteinStability, accessed on 17 July 2025. Additionally, the raw data from Tsuboyama et al. is available at https://doi.org/10.5281/zenodo.7844779, accessed on 21 April 2023. The raw Sarkisyan dataset was acquired from “amino_acid_genotypes_to_brightness.tsv” in the following link https://figshare.com/articles/dataset/Local_fitness_landscape_of_the_green_fluorescent_protein/3102154, accessed on 14 March 2016. The raw data from SKEMPI 2.0 is available at https://life.bsc.es/pid/skempi2/database/index, accessed on 6 June 2018. Code availability: The code used to train ProtT5 models to predict protein stability, GFP brightness, and protein quaternary structure stability and a guide to reproducing the results demonstrated herein is available on our GitHub at https://github.com/ejp-lab/EJPLab_Computational_Projects/tree/master/ProteinStability, accessed on 17 July 2025.

## Supplementary Materials

The following supporting information can be downloaded at: https://www.mdpi.com/article/10.3390/ijms26157125/s1. Reference [31] are cited in the supplementary materials.
