# Supplementary material for: Accurate Prediction of Protein Tertiary and Quaternary Stability Using Fine-Tuned Protein Language Models and Free Energy Perturbation
Source: Int J Mol Sci. 2025 Jul 24;26(15):7125. doi: 10.3390/ijms26157125 (PMC12345697; doi:10.3390/ijms26157125)
Supplement: Supplementary file 1 [file ijms-26-07125-s001.zip › ijms-3761307-supplementary.pdf]

Electronic Supplementary Information

Accurate Prediction of Protein Tertiary and Quaternary Stability Using Fine-Tuned Protein Language Models and Free Energy Perturbation

Xinning Li[1], Ryann Perez[1], John. J. Ferrie[2], E. James Petersson[1]*, and Sam Giannakoulias[1,2]*

[1] Department of Chemistry, University of Pennsylvania, Philadelphia, Pennsylvania 19104, USA

[2] Division for Advanced Computation, Sentauri Inc, Glenwood, Maryland 21738, USA

* Corresponding author

EJP: ejpetersson@sas.upenn.edu; SGG: samgiannakoulias@sentauriai.com

Table of Contents:

1. **Software…………………………………………………………………………………. S2**
2. **Datasets………………………………………………………………………………… S2**
3. **EDA……….……………………………………………………………….…............... S3**
4. **Model Training…………………….…………………………………………………... S11**
5. **FEP……………………….………………………………………………..……………...S13**
6. **References ..……………………………………………………………………………….S14**

Software

The software used for this work can be found in the conda environment yml file alongside installation instructions at the following link: https://github.com/ejp-lab/EJPLab_Computational_Projects/blob/master/ProteinStability/environment.yml.

Datasets

The Tsuboyama dataset was acquired from the following link. https://zenodo.org/record/7401275.

The Sarkisyan dataset was acquired from "amino_acid_genotypes_to_brightness.tsv" in the following link https://figshare.com/articles/dataset/Local_fitness_landscape_of_the_green_fluorescent_protein/3102154.

The Skempi 2.0 dataset was extracted from the following link. https://life.bsc.es/pid/skempi2/database/index

All machine learning datasets were created as described in the main text. For reproducibility, these datasets can be found at our GitHub through the following link: https://github.com/ejp-lab/EJPLab_Computational_Projects/blob/master/ProteinStability.

EDA

The Tsuboyama dataset was then explored thoroughly for potential heuristics which explain stabilization of protein domains. We wrote a Python script which found the average effect of stabilization/destabilization from mutation as a function of amino acid type. Figure S1 displays a bar chart for each unique amino acid mutation type in the dataset.

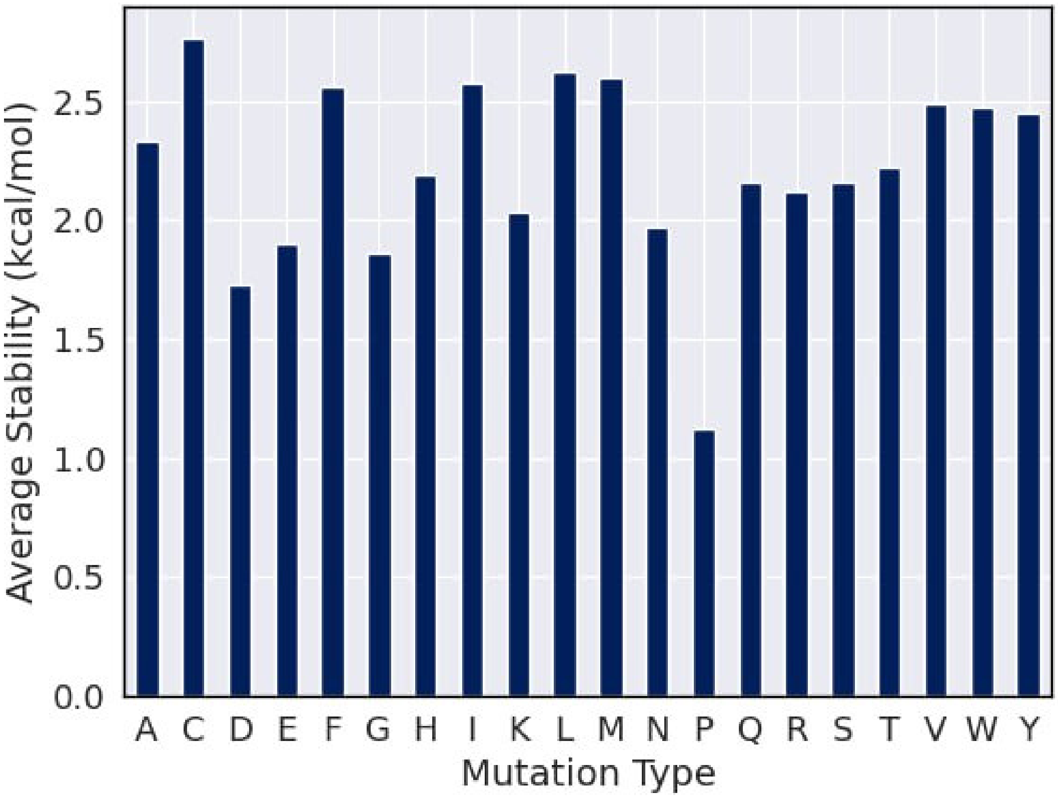

Figure S1. Bar chart plotting average stability in kcal/mol per mutation type in the Tsuboyama dataset.

Seeing as this global analysis largely only provided insight that proline appears as the most destabilizing of the types of mutations, we investigated each of the protein domains individually. Here we found much more informative trends. Figure S2 presents an example bar chart (yeast

Myo5 SH3 domain) where a unique stabilization/destabilization profile is observed relative to the average of the set. The csv titled DomainStatisticsPerMutationType.csv on our GitHub at https://github.com/ejp-lab/EJPLab_Computational_Projects/blob/master/ProteinStability/EDA. It displays the average stabilization/destabilization values for all mutation types in every domain. For clarity, Table S1 provides relevant statistics of this csv file.

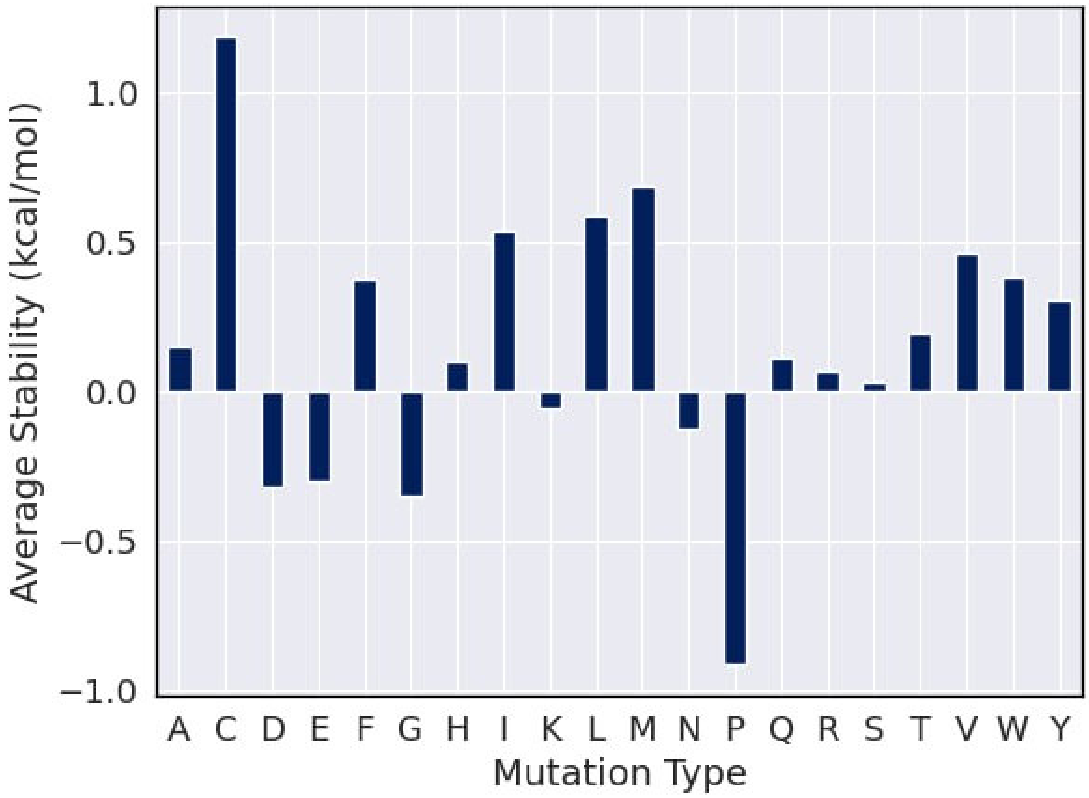

Figure S2. Bar chart plotting average stability in kcal/mol per mutation type in a yeast Myo5 SH3 domain (1YP5).

The same analyses were performed for protein domain secondary structure motifs determined by "define secondary structure of proteins" (DSSP)[1]. Figure S3 displays a bar chart of the global dataset metrics, while Figure S4 shows a unique, representative of a specific domain. Again, we

observed that the global analysis was mostly uninformative, but that individual protein domain analysis reveals many specific trends. Stabilization data for each WT fold can be found at The csv titled DomainStatisticsPerMutationType.csv on our GitHub at https://github.com/ejp-lab/EJPLab_Computational_Projects/blob/master/ProteinStability/EDA, representative metrics for this datasheet can be found in Table S1.

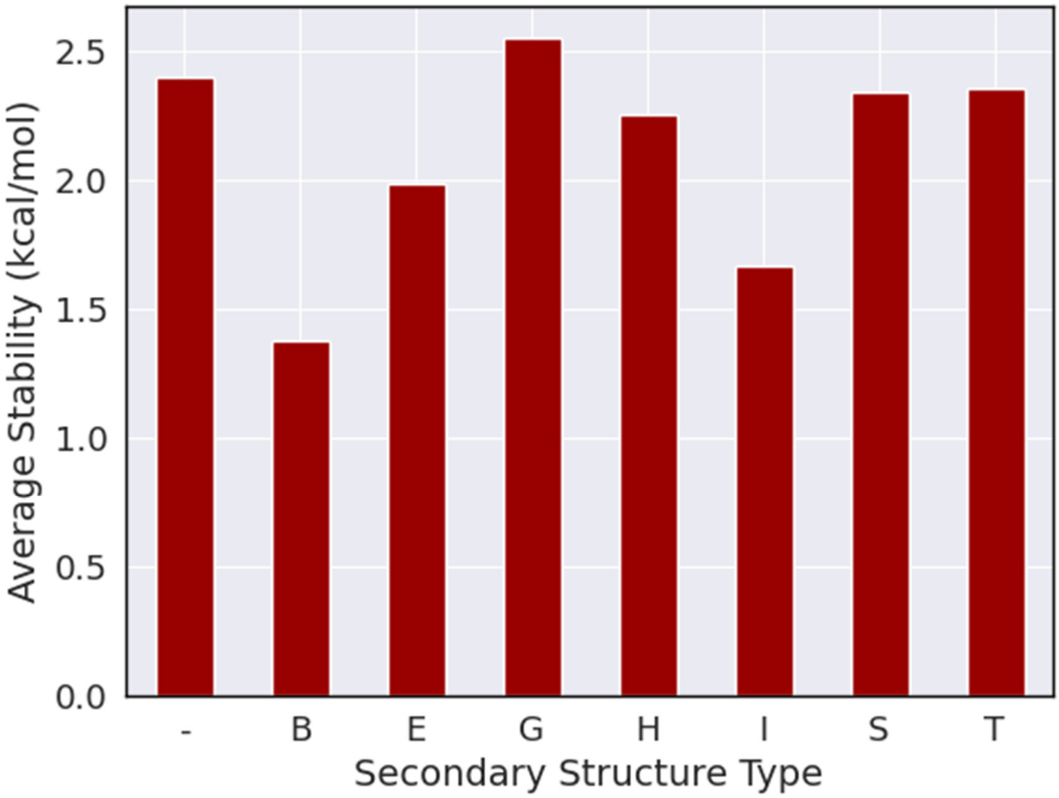

Figure S3. Bar chart plotting average stability in kcal/mol per secondary structure type in the Tsuboyama dataset. Secondary structures: B (residue in isolated β-bridge), E (extended strand in parallel and/or anti-parallel β-sheet), G (3-turn helix), H (4-turn helix), I (5-turn helix). S (bend), and T (hydrogen bonded turn).

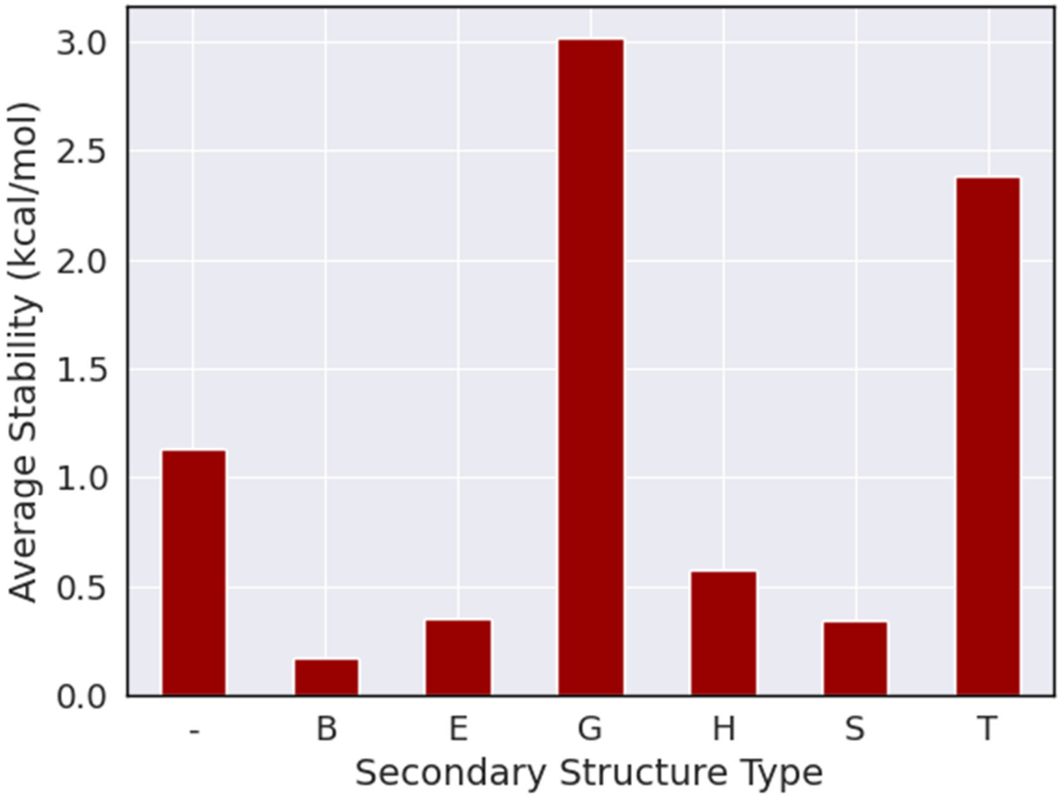

Figure S4. Bar chart plotting stability in kcal/mol per secondary in a type III antifreeze protein (1EKL). Secondary structures: B (residue in isolated β-bridge), E (extended strand in parallel and/or anti-parallel β-sheet), G (3-turn helix), H (4-turn helix), I (5-turn helix). S (bend), and T (hydrogen bonded turn).

Table S1. Table displaying statistics of mutation types and secondary structure* on the Tsuboyama et al dataset.

Mutation Type/ Secondary Structure*	Minimum	Maximum	Mean	Stdev
A	-0.968	4.71	2.314	1.025
C	0.768	4.97	2.751	0.843
D	-0.874	4.187	1.698	0.92
E	-0.81	4.252	1.875	0.929
F	0.342	4.837	2.554	0.963
G	-0.496	4.747	1.842	1.001
H	-0.294	4.558	2.158	0.985
I	-0.256	4.859	2.586	0.954
K	-0.906	4.491	1.977	0.94
L	-0.938	4.851	2.634	0.985
M	0.288	4.976	2.616	0.984
N	-0.469	4.655	1.946	0.98
P	-0.912	3.898	1.094	0.917
Q	-0.395	4.515	2.12	0.957
R	-0.045	4.463	2.094	0.887
S	-0.396	4.837	2.139	0.997
T	-0.043	4.716	2.213	1.006
V	0.203	4.917	2.503	0.981
W	0.372	4.785	2.465	0.923
Y	0.308	4.78	2.439	0.95
-	0.277	4.765	2.365	0.96
B*	-0.315	4.419	1.446	1.19
E*	-0.106	4.61	1.711	1.205
G*	-0.255	4.764	2.264	1.001
H*	-0.477	4.947	1.882	1.269
I*	-0.289	4.847	0.309	0.767
S*	-0.698	4.686	0.79	1.255
T*	0	1.668	0.004	0.078

Mutations refer to changes to the amino acid one letter code listed. *Secondary structures: - (random coil), B (residue in isolated β-bridge), E (extended strand in parallel and/or anti-parallel β-sheet), G (3-turn helix), H (4-turn helix), I (5-turn helix). S (bend), and T (hydrogen bonded turn).

We conducted similar EDA for GFP protein to explain the relationship between brightness and residual mutations. Similarly with the analysis of Tsuboyama dataset, we wrote a Python script which calculated the average effect of protein brightness change from mutation as a function of amino acid type. Figure S5 displays a bar chart for each unique amino acid mutation type in the dataset and its corresponding brightness.

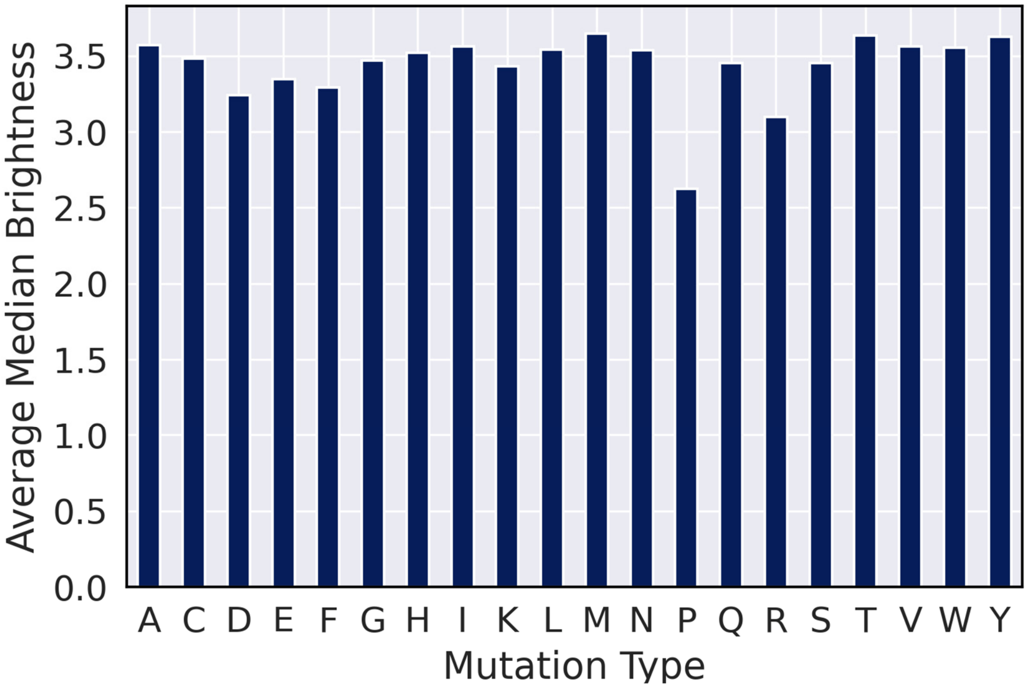

Figure S5. Bar chart plotting average median brightness per mutation type in the Sarkisyan dataset.

This bar plot shows that proline appears as the most influential type of mutation on GFP brightness. To give a better insight into the general trend of brightness as the effect of each mutation type, we provide relevant statistics in Table S2.

The same analyses were performed for secondary structure motifs determined by "define secondary structure of proteins" (DSSP)[1]. Figure S6 displays a bar chart of the average brightness per secondary structure type. In addition, we provide statistics of brightness for secondary structure motifs in Table S2.

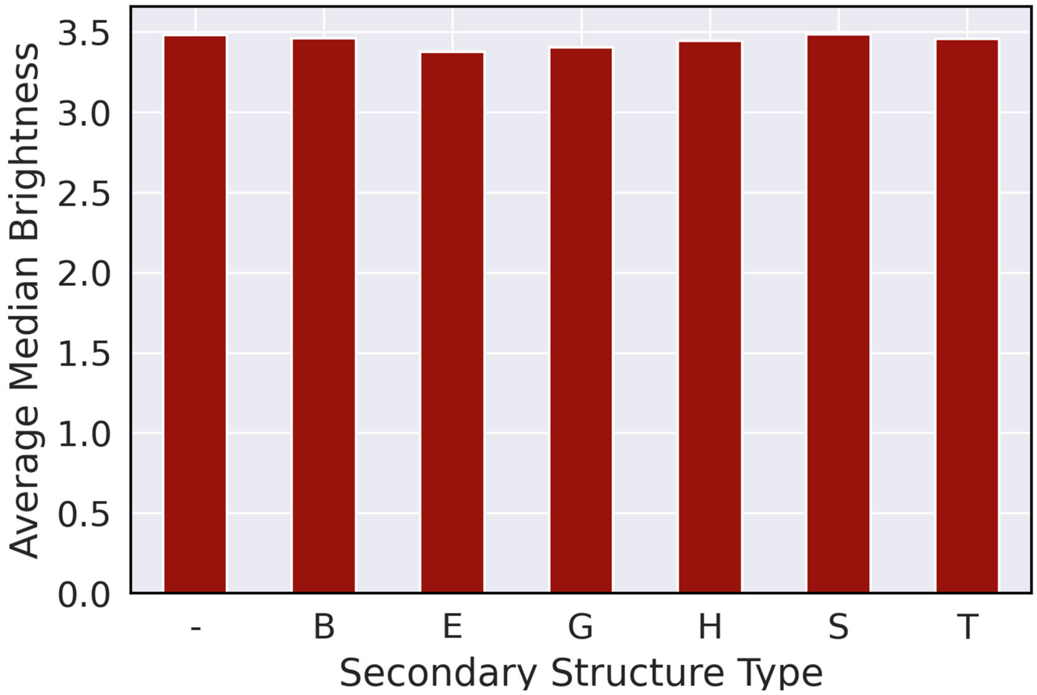

Figure S6. Bar chart of the average brightness per secondary structure type. Secondary structures: B (residue in isolated β-bridge), E (extended strand in parallel and/or anti-parallel β-sheet), G (3-turn helix), H (4-turn helix), I (5-turn helix). S (bend), and T (hydrogen bonded turn).

Table S2. Table displaying statistics of mutation types and secondary structure* on the Sarkisyan et al dataset.

Mutation Type/ Secondary Structure*	Minimum	Maximum	Mean	Stdev
A	1.301	3.833	3.572	0.295
C	1.301	3.855	3.482	0.572
D	1.301	3.771	3.241	0.845
E	1.301	3.874	3.345	0.769
F	1.299	3.786	3.293	0.798
G	1.301	4.114	3.471	0.511
H	1.301	3.895	3.520	0.461
I	1.395	3.834	3.564	0.332
K	1.301	3.746	3.431	0.678
L	1.301	3.992	3.541	0.417
M	3.135	3.829	3.647	0.140
N	1.301	3.863	3.539	0.446
P	1.301	3.785	2.626	1.034
Q	1.301	3.837	3.452	0.625
R	1.301	3.785	3.097	0.976
S	1.301	3.873	3.452	0.584
T	3.122	4.008	3.635	0.166
V	1.301	3.956	3.563	0.451
W	3.380	3.694	3.553	0.159
Y	3.127	3.838	3.625	0.142
-*	1.301	3.907	3.479	0.568
B*	1.401	3.800	3.460	0.606
E*	1.301	4.114	3.377	0.691
G*	1.299	3.992	3.406	0.736
H*	1.528	3.766	3.447	0.548
S*	1.301	3.798	3.485	0.478
T*	1.301	3.804	3.456	0.537

Mutations refer to changes to the amino acid one letter code listed. *Secondary structures: - (random coil), B (residue in isolated β-bridge), E (extended strand in parallel and/or anti-parallel β-sheet), G (3-turn helix), H (4-turn helix), I (5-turn helix). S (bend), and T (hydrogen bonded turn).

Model Training

We fine-tuned our ProtT5 regression and classification models using the pretrained prot_t5_xl_uniref50 from Rostlab[2] in HuggingFace. The final models for each case study were selected from the best trial of Bayesian hyperparameter searching with the TPE sampler in the Optuna Python library[3]. The best parameters for all the models are shown in Table S3.

Ten trials were searched while attempting to minimize the mean squared error loss of the validation set for regression problems, and cross entropy loss for classification tasks. Models were trained for a maximum of 10 epochs with an early stopping patience of 2 epochs. Finally, the tuned models were used to predict the data of the held-out testing set.

Table S3. Best hyperparameters for all models obtained by Optuna Bayesian optimization

	Dropout Weight	Batch Size	Learning Rate	Gradient Accumulation Steps	Lora Rank
ProtT5_ΔG	0.305269907953647	20	0.000445750968802205	2	8
ProtT5_GFP	0.257426929489671	6	0.000437625568455695	5	5
ProtT5_ΔΔG	0.106175348288741	1	0.000970210753640374	12	7

Metrics for validation and test sets

Table S4- 7 are validation and test metrics for Tsuboyama dataset, Sarkisyan dataset, and Skempi 2.0 dataset.

Table S4. Validation and test metrics of ProtT5 fine-tuning on Tsuboyama dataset.

ProtT5_ΔG	Validation	Test
MSE	1.20	1.09

R^2	0.51	0.60

Table S5. Validation and test classification reports of ProtT5 fine-tuning on Sarkisyan dataset.

	Val Precision	Val Recall	Val F1-score	Test Precision	Test Recall	Test F1-score
Dark (class 0)	0.85	0.73	0.79	0.9	0.66	0.76
Bright (class 1)	0.97	0.98	0.97	0.96	0.99	0.97
Macro Avg	0.91	0.86	0.88	0.93	0.83	0.87
Weighted Avg	0.95	0.95	0.95	0.95	0.95	0.95
Overall Accuracy			0.95			0.95

Table S6. Validation and test metrics of ProtT5 fine-tuning on Skempi 2.0 dataset for regression task.

ProtT5_ΔΔG	Validation	Test
MAE	1.18	1.33
R^2	0.49	0.35

Table S7. Validation and test classification reports of ProtT5 fine-tuning on Skempi 2.0 dataset for classification task.

	Val Precision	Val Recall	Val F1-score	Test Precision	Test Recall	Test F1-score
Stabilizing (class 0)	0.55	0.36	0.44	0.3	0.43	0.35
Destabilizing (class 1)	0.82	0.91	0.86	0.86	0.77	0.81
Macro Avg	0.69	0.64	0.65	0.58	0.6	0.58
Weighted Avg	0.76	0.78	0.76	0.75	0.71	0.73
Overall Accuracy			0.78			0.71

FEP

FEP calculations were performed in GROMACS 2024.1 using the Amber 99sb-star-ildn force field and spce water model largely adhering to the standard protocol described by others[4]. In brief, mutations were set up by aligning amino acids to one another based on their maximum common substructure, using RDKit, followed by exhaustive chi angle sampling to generate non-clashed initial poses. Subsequently, dual topologies were generated from these aligned structures and equilibration and 5 ns production MD simulations were performed using the REST2[5] enhanced sampling method across 12 lambda states. Finally, the Bennet Acceptance Ratio (gmx_mpi bar) analysis was performed in GROMACS on the resultant trajectory xvg files to calculate the change in free energy associated with each mutation. Table S8 provides one extra datapoint external to SKEMPI 2.0, an antibody-antigen complex. It shows that FEP can be used in the most common setting for biologics design.

Table S8. FEP simulation results of an antibody antigen complex, external to SKEMPI 2.0.

Mutant	Experimental (kcal/mol)	FEP (kcal/mol)	Error (kcal/mol)
1YY9_C_G30Y	-0.94	-1.05	0.11

References

1. Colloch N, Etchebest C, Thoreau E, Henrissat B, Mornon JP. COMPARISON OF 3 ALGORITHMS FOR THE ASSIGNMENT OF SECONDARY STRUCTURE IN PROTEINS - THE ADVANTAGES OF A CONSENSUS ASSIGNMENT. Protein Engineering. 1993;6(4):377-82. doi: 10.1093/protein/6.4.377. PubMed PMID: WOS:A1993LG32800006.

2. Elnaggar A, Heinzinger M, Dallago C, Rehawi G, Wang Y, Jones L, et al. ProtTrans: Toward Understanding the Language of Life Through Self-Supervised Learning. Ieee Transactions on Pattern Analysis and Machine Intelligence. 2022;44(10):7112-27. doi: 10.1109/tpami.2021.3095381. PubMed PMID: WOS:000853875300088.

3. Akiba T, Sano S, Yanase T, Ohta T, Koyama M, editors. Optuna: A next-generation hyperparameter optimization framework. Proceedings of the 25th ACM SIGKDD international conference on knowledge discovery & data mining; 2019.

4. Wang L, Chambers J, Abel R. Protein–Ligand Binding Free Energy Calculations with FEP. Methods Mol Biol. 20222019. p. 201-32.

5. Wang L, Friesner RA, Berne BJ. Replica Exchange with Solute Scaling: A more efficient version of Replica Exchange with Solute Tempering (REST2). J Phys Chem B. 2011;115(30):9431-8. doi: 10.1021/jp204407d PMID - 21714551 PMCID - PMC3172817.
